# Our Evolving Understanding of Kawasaki Disease Pathogenesis: Role of the Gut Microbiota

**DOI:** 10.3389/fimmu.2020.01616

**Published:** 2020-07-24

**Authors:** Kazunari Kaneko, Shohei Akagawa, Yuko Akagawa, Takahisa Kimata, Shoji Tsuji

**Affiliations:** Department of Pediatrics, Kansai Medical University, Osaka, Japan

**Keywords:** Kawasaki disease, regulatory T cell, T helper 17 cell, allergy, gut microbiota, dysbiosis

## Abstract

Kawasaki disease (KD) was first described by Dr. Tomisaku Kawasaki in 1967. The etiology of KD has been studied comprehensively but remains largely unknown. The disease seems to result from the interplay of genetic and environmental susceptibility factors with infectious triggers, followed by a subsequent abnormal immune response characterized by increased levels of inflammatory cytokines and chemokines during the acute phase. Evidence has mounted to suggest that an imbalance between T helper 17 cells (Th17s) and regulatory T cells (Tregs) is associated with aberrant immune responses in KD. Recent advances in culture-independent techniques for detection and identification of intestinal commensal bacteria enabled the discovery that Th17 and Treg differentiation are regulated by short chain fatty acids (SCFAs), in particular butyrate, produced by the gut microbiota. This finding provided a mechanistic link between dysbiosis, defined as changes in the composition of the gut microbiota, and various inflammatory diseases. On this basis, we propose that dysbiosis, with reduced production of SCFAs leading to imbalances of Th17s/Tregs, could be involved in the etiology of KD. A pilot study supported this hypothesis, as only fecal concentrations of butyrate were significantly reduced in KD patients among SCFAs. This evolving perspective prompted us to undertake metagenomic analyses of bacterial DNA from the feces of KD patients who were antibiotic-naïve at diagnosis. Simultaneous measurements of Th17s/Tregs in peripheral blood and SCFA concentrations in feces would provide valuable information regarding the association between dysbiosis and dysregulated immune responses in KD.

## Introduction

Kawasaki disease (KD), named after Dr. Tomisaku Kawasaki deceased June 5th, 2020, mainly affects young children between the ages of 6 months and 4 years (1). KD is characterized by persistent fever, bilateral conjunctival congestion, changes of the lips and oral cavity, polymorphous exanthema, changes of peripheral extremities, and acute non-purulent cervical lymphadenopathy (2, 3). Although KD was originally reported to be self-limiting and benign (4), it is now recognized as a systemic vasculitis with a specific predilection for forming coronary artery lesions. These develop in up to 25% of children with KD who are not treated with intravenous immunoglobulin (5). Coronary artery lesions associated with KD are the most common causes of pediatric heart disease in developed countries. The incidence of KD in the Japanese population continues to increase and reached 330 cases per 100,000 children aged ≤4 years per year in 2015 (6).

Despite extensive ongoing research into the etiology of KD, the underlying mechanisms of this enigmatic vasculitis are not fully understood (3, 7).

## Current Paradigm of the Etiology of KD

The current paradigm of KD pathogenesis is that the disease results from a pathologically amplified immune response against infectious agent(s) in a genetically and environmentally susceptible child (3). This paradigm is based on the following observations.

### Proposed Infectious Causes of KD

First, there is clinical overlap between KD and infectious diseases such as adenovirus and streptococcosis. Second, seasonal clustering of KD in the winter and spring mimics that of several viral diseases (8). Third, temporal clusters of epidemics have been reported in Japan, the US, Canada, and Finland (9). Moreover, an outbreak in Japan began in Tokyo and spread throughout the country over a period of 6 months (10). Finally, low incidence in the first 3 months of life suggests at least partial protection from trans-placental antibodies (11). The low incidence of KD in schoolchildren indicates a potential role of common antigen(s) that most children encounter uneventfully in early childhood and against which they mount an appropriate and protective immune response (12). However, efforts to find a single unifying microbiological cause of KD have been, to date, unsuccessful. Standard microbiological techniques, molecular methods and serological investigations have all failed to identify an etiological agent.

It has long been held that infection by one or more widely distributed microorganism(s) might elicit dysregulated immune responses in genetically susceptible children resulting in KD. Candidate pathogens include Epstein-Barr virus (13, 14), human herpes virus (15), human immunodeficiency virus (16), human adenovirus (17), human coronavirus (18), retrovirus (19), human parvovirus B19 (20), human bocavirus (21), S*taphylococcus aureus* (22), *Streptococcus pyogenes* (23)*, Yersinia pseudotuberculosis* (24)*, Bacillus cereus* (25)*, Mycoplasma pneumoniae* (26)*, Mycobacterium spp*. (27)*, Bartonella henselae* (28), *Coxiella burnetti* (29), and *Candida* spp. (30). *Candida albicans* (*C. albicans*) has recently drawn attention, as administration of CAWS (water-soluble extracellular polysaccharide from culture supernatants of *C. albicans*) induces coronary arteritis similar to KD in mice (31). Several reports suggested that *C. albicans* plays an important role as an infectious trigger of KD (30, 32, 33). Considering the recent pandemic of coronavirus disease 2019 (COVID-19), potential links between KD and COVID-19 are also deserving of mention (34–37). Clusters of children presenting with KD-like symptoms have been documented in the UK, US, France, and Italy, and some of them were confirmed to have COVID-19. It appears that hyperinflammation associated with COVID-19 could act as primer for KD development in individuals having genetically or environmentally determined predisposition. However, the specific mechanism is not yet defined (34).

### Genetic and Environmental Risk Factors for KD

The higher incidence of KD in Asian countries suggests a genetic predisposition for acquiring the disease (9). Increased risk in family members of KD patients in Japanese populations (38) also hint at genetically determined susceptibility. Recent advances have been made in identifying disease-susceptibility genes from genome-wide association studies. Candidate genes contributing to KD susceptibility include B-lymphoid kinase (*BLK*) (39), caspase-3 (*CASP3*) (40), low-affinity immunoglobulin gamma Fc region receptor II-a (*FCGR2A*) (41), human leukocyte antigen (*HLA*) (39), inositol 1,4,5-triphosphate kinase-C (*ITPKC*) (42), transforming growth factor (TGF)-β2 (*TGF*β*2*) (43), TGF-β receptor 2 (*TGFBR2*) (43), *SMAD3* (43), *CD40* (39), and *ORAI1* (44).

Epidemiologic studies have extensively searched for environmental factors that may explain variation and seasonality in KD incidence. Surveys from the US and Japan have demonstrated that higher precipitation and lower temperatures were associated with higher incidence of KD (45, 46). An investigation of the role of the early social environment in KD susceptibility in a Japanese population found that higher household income, smaller family size, and urbanization were associated with increased KD incidence (47). This study, however, did not find a significant association between absence of infectious exposures during early life and KD.

### Aberrant Immune Responses in KD

The prominent role played by the immune system in KD has been confirmed by many studies demonstrating activation of neutrophils and other immune cells as well as overproduction of inflammatory cytokines and chemokines such as tumor necrosis factor-α, interleukin (IL)-1, IL-2, IL-6, and IL-8, and monocyte chemotactic protein-1 (3). Levels of inflammatory cytokines and chemokines are reported to be elevated during the acute phase of KD. However, the mechanisms responsible for abnormal immune responses and overexpression of inflammatory cytokines remain unclear. Several lines of evidence have revealed decreased numbers of regulatory T cells (Tregs) and imbalances between T helper 17 cells (Th17s) and Tregs in acute KD (48–50). Jia et al. elegantly demonstrated that Th17 proportions and cytokine (IL-17, IL-6, and IL-23) levels were significantly increased, while Treg proportions and expression of Treg transcription factors (e.g., FoxP3) were significantly decreased in patients with acute KD (49). They concluded that Th17 expansion and Treg depletion were characteristic of acute KD. Furthermore, recent studies suggested that treatment efficacy was associated with decreased Th17 proportions and increased Treg proportions, with both returning closer to a normal range (48, 51). Thus, Th17/Treg imbalances may contribute to exaggerated immune responses in KD patients.

## The Importance of Gut Microbiota in Human Health

Growing evidence suggests that disturbances within intestinal communities of commensal bacteria may lead to illness through aberrant immune system development. While the study of gut microbiology related to human health has a centennial history, recent technological advances have enabled us to explore this field in a more sophisticated manner. Current approaches rely primarily on culture-independent methods such as amplification of conserved regions of the 16S rRNA gene present in all bacteria (52). Studies using these techniques have demonstrated that an adult humans harbor 100 trillion gut bacteria comprising more than 1,000 different species and approximately 160 species per person per fecal sample (53). Development of the gut microbiota, defined as its colonization by microorganisms, might begin not at birth but *in utero*. However, the existence of viable bacteria in the womb was recently questioned (54–56). The maternal microbiota provides the first microbial inoculum, and from birth, microbial diversity increases and converges toward an adult-like microbiome within the first 3–5 years of life (53). The composition of the microbiota in childhood depends on various factors including sanitation, mode of delivery, maturity at birth, infant diet, antibiotic use during infancy, immunizations, and environmental factors such as geography or diet (52, 53, 57, 58). Ethnic and genetic factors may also give rise to dysbiosis (59, 60). The factors that can alter the microbiome are being studied as potential drivers of changing trends in non-communicable diseases. Dysbiosis, defined as changes in the composition of the gut microbiota, may be associated with several clinical conditions including obesity and metabolic diseases (61), cancer (62), autoimmune diseases (63), allergy (64), chronic inflammatory bowel diseases (65, 66), chronic kidney diseases (67), and autistic-spectrum disorders (68). We also found that dysbiosis was present in children with idiopathic nephrotic syndrome (69).

Although research on the mechanism(s) through which dysbiosis impairs human health has just begun, regulation of the gut immune system by microbiota is believed to be involved. Experiments with germ-free animals (deficient in commensal microbiota including gut microbiota) have demonstrated that microbial colonization promotes anatomical development of the intestinal epithelium, increases epithelial cell turnover rates, and initiates the maturation of gut-associated lymphoid tissue (70). Both Tregs and Th17s have received much attention in terms of their role in the gut immune system and its regulation by microbiota (71). Tregs were originally identified as CD4-positive, CD25-positive and Foxp3-positive T cells that exerted inhibitory control over immune responses (72), maintained tolerance to self-antigens, and prevented autoimmune disease (73). There are several immunosuppressive mechanisms mediated by Tregs, including secretion of immunosuppressive cytokines such as IL-10 and TGF-β, functional modification or killing of antigen-presenting cells, and cell contact-dependent suppression via cytotoxic T-lymphocyte- associated protein 4 (72). Tregs mainly arise from naïve T cell precursors following stimulation by short chain fatty acids (SCFAs) such as acetate, propionate or butyrate produced by the gut microbiota (74, 75). Previous studies demonstrated that members of the genus *Clostridium* were potent inducers of Treg differentiation through butyrate production (76, 77) and that reduced luminal concentrations of SCFAs resulted in impaired development of intestinal Tregs in germ-free mice (74, 76). In these mice, reconstitution with commensal bacteria or administration of SCFAs, especially butyrate, restored Treg frequency (76), supporting the role of bacterial metabolites in Treg development. Therefore, it has been proposed that a decrease in the relative abundance of butyrate-producing microbes may disrupt mucosal immune homeostasis (78). The gut microbiota also plays a crucial role in the induction of effector T cell responses in the intestine. Th17s are a subtype of CD4-positive T cells specialized for mounting immune responses against fungi and some extracellular bacteria. In addition to IL-17A, Th17s produce IL-17F, IL-21, IL-22, and IL-26 (79). Because IL-17 is a potent proinflammatory cytokine that amplifies ongoing inflammation, aberrant regulation of Th17s contributes to development of inflammatory and autoimmune disorders. In germ-free mice, the number of Th17s was markedly decreased. However, the Th17 compartment was restored by reconstitution with conventional microbiota (80), thus indicating a crucial role for gut microbes in Th17 development. Among commensal bacteria, segmented filamentous bacteria (SFB) are one of the most potent inducers of Th17s. Colonization of mice by SFB causes abundant accumulation of Th17s in the small intestine via enhanced production of serum amyloid A (81, 82). The existence of human commensal bacteria equivalent to SFB in rodents is probable because mixtures of bacterial strains isolated from fecal samples ulcerative colitis patients could induce Th17 development (81).

## The Gut Microbiota in KD

The gut, the largest interface between microbial factors and the host, contains the largest proportion of bacteria and the largest amount of lymphoid tissue in the body. Thus, it was hypothesized that the intestinal environment might be reshaped in patients with KD. Indeed, KD patients frequently exhibit gastrointestinal symptoms and complications (83). The contribution of the gut microbiota to KD has been evaluated in limited numbers of small cohorts using culture-based methods. Several studies have been performed to identify the causative microbial agent(s) of KD at disease onset. Takeshita et al. showed that the gut microbiomes of KD patients were distinguished by a lack of *Lactobacilli* during the acute phase (84), while Nagata et al. isolated both HSP60-producing Gram-negative bacteria and Gram-positive cocci capable of inducing Vβ2 T cell expansion from KD patients (85). These results indicated that distinctive gut microbes might be involved in the pathophysiology of KD. However, because these studies of the microbiomes of KD patients were carried out using culture-based methods, microorganisms that cannot be cultured, which constitute more than half of the human gut microbiome, would have been overlooked.

Unlike culture methods, metagenomic analyses can reveal the composition of the intestinal microbiota irrespective of the ability to culture microbes. Kinumaki et al. first reported the results of a metagenomic analysis of feces using culture-independent methods (86). They collected 28 paired fecal samples from children with KD during the acute and non-acute phases and demonstrated that *Streptococcus* spp., including *S. pneumoniae, S. pseudopneumoniae, S. mitis, S. oralis, S. gordonii*, and *S. sanguinis*, were more abundant during the acute phase while the genera *Ruminococcus, Roseburia* and *Faecalibacterium* were less abundant. Unfortunately, more than half of subjects were treated empirically with antibiotics during the early phase of KD when the fecal samples were collected because clinical and laboratory findings often do not fulfill the diagnostic criteria of KD (2), but are instead suggestive of bacterial infections (87). As antibiotic administration rapidly perturbs the gut microbiota (88), these results may reflect the effects of antibiotic therapy on the gut microbiota and not dysbiosis associated with KD.

## Novel Perspectives on KD Pathogenesis

**Figure 1:**
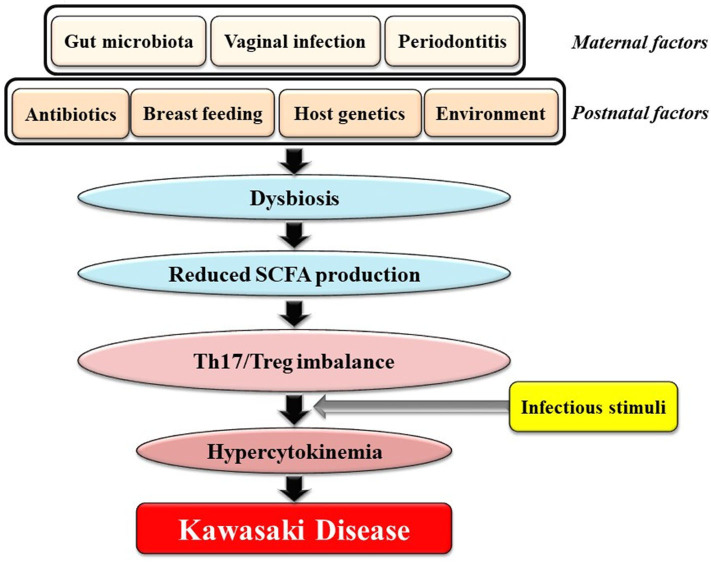
Potential association between gut dysbiosis and Kawasaki Disease (KD). Various factors during both the *in utero* and postnatal period could drive dysbiosis in infants. Dysbiosis results in reduced intestinal production of SCFAs including butyrate. Reduced SCFA concentrations cause an imbalance of Th17s/Tregs. Individuals with Th17/Treg imbalances develop hypercytokinemia following stimuli from one or more infectious agents(s) followed by KD. SCFAs, short chain fatty acids.

Here, we would like to focus on a novel viewpoint on the role of the gut microbiota in KD: *Dysbiosis, defined as changes in the composition of the gut microbiota and caused by various prenatal and postnatal factors that are not necessarily infectious agent(s), might contribute to genetically and environmentally determined predilection for KD*. This perspective is illustrated in Figure 1 and can be explained as follows: [1] various factors during the *in utero* and postnatal period drive dysbiosis in young children; [2] dysbiosis results in reduced intestinal production of SCFAs including butyrate; [3] reduced levels of SCFAs in the gut cause an imbalance of Th17s/Tregs; and [4] individuals with Th17/Treg imbalances develop hypercytokinemia triggered by ubiquitous infectious agents(s), followed by KD (Figure 1). Recent observations revealed that viral respiratory infections may alter microbial growth in the gut leading to dysbiosis (89). In addition, the gut microbiota has been shown to play an important role in regulating the generation of virus-specific CD4-positive and CD8-positive T cells and antibody responses following influenza virus infection (90). Therefore, we hypothesize that young children with dysbiosis are prone to a vicious cycle of hypercytokinemia following infection by viruses.

This paradigm for the pathogenesis of KD contrasts with previous hypotheses, which focused on specific microorganisms, toxins, or pathogen-associated molecular patterns (91–93).

As butyrate has been reported to limit Th17 differentiation and promote Treg development (94–96), we hypothesize that KD-associated dysbiosis might be characterized by lower abundance of butyrate-producing bacteria. Interestingly, the genera *Roseburia* and *Faecalibacterium*, which were reported to be less abundant in patients with acute KD by Kinumaki et al. (86), are butyrate-producing bacteria (97, 98). Furthermore, the strong association between KD and allergic diseases (99–101) in which dysbiosis plays an important role (102) also supports this perspective. Recent observations of a potential association between previous antibiotic therapy and development of KD also supports our hypothesis (103). In this study, the median interval between the final dose of antibiotics and the onset of KD was 2.5 months, which was insufficient time for restoration of the gut microbiota and complete resolution of dysbiosis caused by antibiotic use (104).

To confirm our hypothesis, further investigations involving metagenomic analysis of bacterial DNA from the feces of a larger number of antibiotic-naïve patients with KD is clearly needed. It would be worthwhile to compare the proportions of specific microbial species such as butyrate-producing bacteria. Simultaneous analysis of Th17/Treg ratios in peripheral blood and measurement of fecal butyrate concentrations, reflecting the intestinal production of SCFAs, would also be helpful.

We have just begun a project to test our hypothesis with approval from our institutional ethics committee (approval no. 2015127) and parental informed consent. Fecal samples will be collected not only from KD patients and healthy children but also from controls with viral infections. This will allow us to specifically characterize dysbiosis in KD because recent observations suggested that viral infection itself may cause dysbiosis (89).

**Figure 2:**
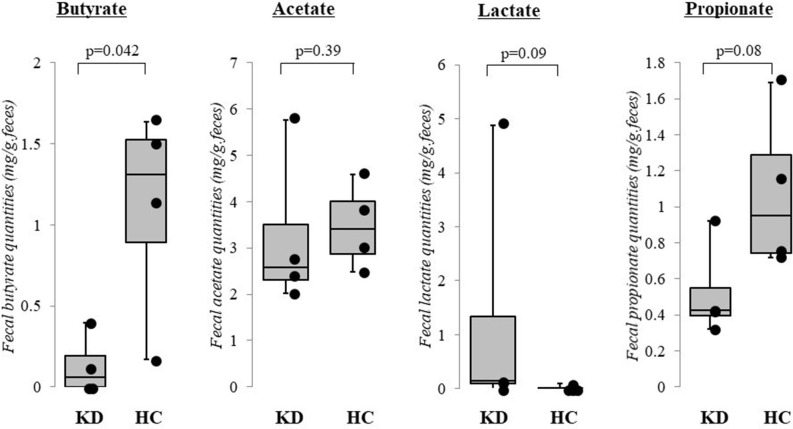
Results of pilot study: fecal organic acid concentrations in Kawasaki disease (KD). Fecal butyrate concentrations were significantly lower in KD patients (*p* < 0.05, Mann–Whitney *U* test) while concentrations of acetate, lactate, and propionate did not differ between KD patients and healthy control children. KD: Kawasaki disease; HC: age-matched healthy children. The horizontal lines in the boxes represent the median values, and the bottoms and tops of the boxes represent the 25th and 75th percentiles, respectively. The vertical lines extend from the box to the maximum values and minimum values. Frozen fecal specimens were thawed, and a 0.1 g subsample was placed in a 2.0 mL tube with zirconia beads and suspended in 0.1 mM perchloric acid solution containing 3% phenol. Samples were heated at 80°C for 15 min, vortexed at 5 m/s for 45 s using FastPrep 24 instrument (MP Biomedicals, Irvine, CA, USA), and centrifuged at 15,350 g for 10 min. Supernatants were filtered using 0.45 μm filters. Fecal organic acids including acetate, propionate, butyrate, and lactate were measured using high-performance liquid chromatography (Prominence, Shimadzu, Kyoto, Japan) using a post-column reaction with a detector (CDD-10A, Shimadzu), two columns arranged in tandem (Shim-pack SCR-102(H), 300 × 8 mm ID, Shimadzu) and a guard column (Shim-pack SCR-102(H), 50 × 6 mm ID, Shimadzu). The mobile phase was 5 mM p-toluenesulfonic acid and the reaction solution was 5 mM p-toluenesulfonic acid containing 100 μM ethylenediaminetetraacetic acid and 20 mM Bis-Tris. The flow rate and oven temperature were 0.8 mL/min and 45°C, respectively. The detector cell temperature was kept at 48°C. Measurements were performed at Techno Suruga Lab, Shizuoka, Japan.

The results of our pilot study of four acute KD patients (median age 1.1 years, range 0.8–2.1 years; 3 boys and 1 girl) and four healthy children (median age 2.1 years, range 1.2–3.7 years, 3 boys and 1 girl) supports our perspective as fecal butyrate concentrations were significantly lower in KD patients (*p* < 0.05, Mann–Whitney *U* test). In contrast, fecal concentrations of acetate, lactate, and propionate did not differ between KD patients and healthy children (Figure 2). The KD patients studied had a median body mass index of 14.7 (range: 13.0–18.1), median maximal body temperatures of 39.0°C (range: 38.0–39.7°C), and median maximal C-reactive protein levels of 41 mg/L (range: 20–57 mg/L). All KD patients presented with typical clinical features and fulfilled the diagnostic criteria (2). None reported diarrhea or constipation and none received antibiotics. Fecal samples were provided before intravenous immunoglobulin administration. No cases were complicated by coronary artery lesions.

What factors perturb the gut microbiota and cause dysbiosis in young children? As shown in Figure 1, both prenatal and postnatal conditions affect the establishment of the intestinal microbiota in infancy. Factors that influence the initial colonization of the gut by microbes include maternal factors such as the maternal gut microbiota, vaginal infection or periodontitis as well as postnatal factors such as cesarean delivery, formula feeding, excessive antibiotic use, host genetics, and the environment (105). Interestingly, formula feeding (106) and social environment factors such as higher household income, smaller family size, and urbanization were associated with both increased risk of dysbiosis and increased KD incidence (47). In addition, the peak age of KD onset ranging from 6 months to 4 years corresponds to the critical period for establishment of the gut microbiota during the first 1,000 days of life (107).

## Conclusions

We believe that dysbiosis underlies KD and could contribute to genetically and environmentally determined predilections for KD. Therefore, KD might be included in the growing list of dysbiosis-associated conditions. If our perspective is confirmed, it would be valuable to investigate whether supplying probiotics starting at birth could reduce the risk of KD in infancy.

## Data Availability Statement

The raw data supporting the conclusions of this article will be made available by the authors, without undue reservation.

## Ethics Statement

The studies involving human participants were reviewed and approved by Ethics Committee of Kansai Medical University. Written informed consent to participate in this study was provided by the participants' legal guardian/next of kin.

## Author Contributions

All authors listed have made a substantial, direct and intellectual contribution to the work, and approved it for publication.

## Conflict of Interest

The authors declare that the research was conducted in the absence of any commercial or financial relationships that could be construed as a potential conflict of interest.
